# Editorial: Inflammation in Obesity: From Physiological to Pathological Aspects

**DOI:** 10.3389/fnut.2022.870131

**Published:** 2022-03-25

**Authors:** Marina C. Oliveira, Geneviève Marcelin, Emmanuel L. Gautier, Adaliene V. M. Ferreira

**Affiliations:** ^1^Immunometabolism, Department of Nutrition, Nursing School, Universidade Federal de Minas Gerais, Belo Horizonte, Brazil; ^2^INSERM, Nutrition and Obesities, Systemic Approach (NutriOmics) Research Unit, UMRS U1269, Sorbonne Université, Paris, France; ^3^Sorbonne Université, INSERM UMR-S 1166, Hôpital de la Pitié-Salpêtrière, Paris, France

**Keywords:** obesity, inflammation, metabolism, diet, chronic inflammatory disease

Obesity is a worldwide disease characterized by adipose tissue expansion and remodeling associated with metabolic dysfunction and chronic low-grade inflammation. The expansion of the adipose mass in obesity can occur through increased adipocyte volume (hypertrophy) and/or increased adipocyte numbers (hyperplasia). Together with fat mass growth, inflammatory mediators, such as pro-inflammatory cytokines (TNF, IL-6, and IL-1b), are released during obesity, while anti-inflammatory mediators (adiponectin, IL-10) are reduced. At early stages of the obesogenic process, inflammation appears as a physiological response that helps to maintain metabolic and energy homeostasis. However, when obesity enters its chronic phase, adipocyte hypertrophy associated with vasculature dysfunctions lead to suboptimal tissue perfusion and local hypoxia. This favors adipocyte demise and leads to local and systemic low-grade inflammation. In a persistent obesogenic environment, recruitment and activation of macrophages can promote systemic insulin resistance through the release of pro-inflammatory cytokines, such as TNF and IL-6, that stimulate lipolysis in adipocytes and favor the release of free fatty acids. Such inflammatory-mediated remodeling of the adipose tissue then precipitates the development of obesity comorbidities, including cardiovascular diseases, type 2 diabetes, fatty liver, and some types of cancer.

In this issue, we aim to discuss the pathophysiological aspects of inflammation during the development of obesity. On one side, balanced inflammation can control adipose tissue expansion and appropriately maintain metabolic homeostasis. On the other side, overt inflammation eventually leads to adipose tissue maladaptive remodeling and obesity's dysfunctions. In this context, better defining the molecules and inflammatory pathways that may protect or worsen the metabolic and endocrine systems will help advance to design of preventive or therapeutic actions to combat obesity.

The present Research Topic provides a collection of high-quality manuscripts presenting different aspects of obesity and its interaction with inflammation. This issue comprises six manuscripts, including five original research articles and one review.

Two original articles, from Lopez-Perez et al. and Rohm et al., focus on immune cells alterations in obese patients. The first study assessed the number of mast cells in the omental and subcutaneous adipose tissue. Minor changes in the glycemic control, considering T2D patients, reduced the number of mast cells in white adipose tissue (WAT) and its capacity, especially omental WAT, to store lipids and cause hypoxic cell deaths that will trigger inflammation. In the second study, Rohm et al. characterize several macrophage populations along the gastrointestinal tract and conclude that gut inflammation and accumulation of pro-inflammatory intestinal macrophagesis increased in obese patients.

In animal models, Kovačević et al. demonstrated the harmful effect of dietary fructose on inflammation and insulin signaling in visceral adipose tissue (VAT) of female and male adult rats, evaluating sex-related differences in susceptibility and progression of metabolic alterations. They suggest that VAT inflammation could precede obesity and start even before a measurable increase of VAT mass, making it a silent risk factor for related metabolic disorders.

Two other studies demonstrated how treating obesity with a dietary supplement could healthily modulate inflammation. Jin et al. showed the mechanism by which the dietary betaine regulates the lipid metabolism and inflammation in juvenile black seabream (*Acanthopagrus schlegelii*) fed a high-fat diet. Dietary betaine attenuates hepatic steatosis and inflammatory responses through the Sirt1/Srebp-1/Ppar*A* pathway. Also, Ramadhin et al. examined the anti-inflammatory effects of two human milk oligosaccharides (P3DEX and NTDEX) in high-fat diet induced obesity. Comparing the effects of P3DEX and NTDEX, they concluded that minor structural differences significantly affect the conjugates' therapeutic abilities. Indeed, while P3DEX improved metabolic alterations, WAT inflammation and hepatic lipid accumulation, NTDEX did not ameliorate these parameters or even worsened the phenotype.

Lastly, the review of Russo et al. discusses how the metabolic reprogramming of macrophages can be influenced by changes in the microenvironment induced by metabolic dysfunction typical of obesity and type 2 diabetes. They notably mention the possibility that intra- and extracellular levels of certain metabolites could help to better identify subsets of polarized macrophages that are unique in both diseases. They also describe the metabolic changes that occur intracellularly during macrophage activation.

Overall, the studies presented in this Research Topic focus on different aspects of inflammation in the context of obesity and comorbidities associated, considering animal models and human beings. The studies showed the relevance of the inflammatory response in the modulation of obesity and its impact on health and brought cues to the use of dietary components as potential tools to treat obesity.

## Author Contributions

All authors listed have made a substantial, direct, and intellectual contribution to the work and approved it for publication.

## Funding

We acknowledge support from the Fondation pour la Recherche Médicale, the French National Agency of Research (Adipofib, Captor, Macburn programs), the research program CAPES-COFECUB (Coordenação de Aperfeiçoamento de Pessoal de Nível Superior-Comité Français d'Évaluation de la Coopération Universitaire et Scientifique avec le Brésil) grant number 88887.130206/2017-01, Fundação de Amparo à Pesquisa do Estado de Minas Gerais (FAPEMIG), Conselho Nacional de Desenvolvimento Científico e Tecnológico (CNPq), the European Foundation for the Study of Diabetes, and the Société Française de Nutrition, the Association Française d'Étude et de Recherche sur l'Obésité.

## Conflict of Interest

The authors declare that the research was conducted in the absence of any commercial or financial relationships that could be construed as a potential conflict of interest.

## Publisher's Note

All claims expressed in this article are solely those of the authors and do not necessarily represent those of their affiliated organizations, or those of the publisher, the editors and the reviewers. Any product that may be evaluated in this article, or claim that may be made by its manufacturer, is not guaranteed or endorsed by the publisher.

